# Development of Neighborhood Trajectories employing Historic Redlining and the Area Deprivation Index

**DOI:** 10.21203/rs.3.rs-3783331/v1

**Published:** 2023-12-22

**Authors:** Heather Carlos, Julie E. Weiss, Benjamin Carter, Ellesse-Roselee L. Akré, Adrian Diaz, Andrew P. Loehrer

**Affiliations:** Dartmouth Cancer Center; Dartmouth Cancer Center; The Dartmouth Institute for Health Policy and Clinical Practice; Johns Hopkins Bloomberg School of Public Health: Johns Hopkins University Bloomberg School of Public Health; The Ohio State University Department of Surgery; Dartmouth Hitchcock Medical Center Department of Surgery

## Abstract

The role of historic residential redlining on health disparities is intertwined with policy changes made before and after the 1930s that influence current neighborhood characteristics and shape ongoing structural racism in the United States. We developed Neighborhood Trajectories which combine historic redlining data and the current neighborhood socioeconomic characteristics as a novel approach to studying structural racism.

Home Owners Loan Corporation (HOLC) neighborhoods for the entire U.S. were used to map the HOLC grades to the 2020 U.S. Census block group polygons based on the percentage of HOLC areas in each block group. Each block group was also assigned an Area Deprivation Index (ADI) from the Neighborhood Atlas^®^. To evaluate changes in neighborhoods from historic HOLC grades to present degree of deprivation, we aggregated block groups into “Neighborhood Trajectories” using historic HOLC grades and current ADI. The Neighborhood Trajectories are “Advantage Stable”; “Advantage Reduced”; “Disadvantage Reduced”; and “Disadvantage Stable.”

Neighborhood Trajectories were established for 13.3% (32,152) of the block groups in the U.S., encompassing 38,005,799 people. Overall, the Disadvantage-Reduced trajectory had the largest population (16,307,217 people). However, the largest percentage of Non-Hispanic/Latino Black residents (34%) fell in the Advantage-Reduced trajectory, while the largest percentage of Non-Hispanic/Latino White residents (60%) fell in the Advantage-Stable trajectory.

The development of the Neighborhood Trajectories affords a more nuanced mechanism to investigate dynamic processes from historic policy, socioeconomic development, and ongoing marginalization. This adaptable methodology may enable investigation of ongoing sociopolitical processes including gentrification of neighborhoods (Disadvantage-Reduced trajectory) and “White flight” (Advantage Reduced trajectory).

## Background

Structural and systemic factors are central to ongoing racial and socioeconomic inequities in the United States^1–3^. Residential location is one such structural factor that influences a range of social, economic, and health-related outcomes. Increasing attention is being given to understanding the influence of residential location on a range of health and health care outcomes. However, many methods used to study historic or current neighborhood characteristics fail to fully capture the dynamic aspects of how neighborhoods influence health-related outcomes^4, 5^. For example, wealthy neighborhoods in present day may have been historically affluent, accumulating wealth over time, or due to a recent transfer of wealth as the result investment, development, and the displacement of poorer residents in these communities. Similarly, the racial composition of neighborhoods has been historically shaped by explicit public policies and private practices of landlords, realtors, lending companies^6, 7^. Some current neighborhood compositions are the results of the residual effects of legalized residential segregation while others were altered through gentrification, a neighborhood change processes which tends to displace the current residents that often are people of color and replace them with wealthier and/or White populations^8, 9^. These dynamic aspects of communities shape the structure, systems, and interpersonal interactions beyond either the historic or current composition in isolation. To study these dynamics, we developed an approach called “Neighborhood Trajectories” to facilitate our understanding of how changing neighborhood environmental characteristics may influence current realities.

Neighborhood Trajectories combine historic attributes with current indices into classifications that capture the lasting or changing make-up of a community. Our starting point was historic maps from the 1930’s and 1940’s of urban neighborhoods across the United States (U.S.) that capture grading commonly known as “redlining.” Redlining originated with The Home Owners’ Loan Act of 1933 with the primary goal of providing government-backed residential mortgages to boost home ownership during the Great Depression.^10^ The Home Owners’ Loan Corporation (HOLC) then graded neighborhoods in hundreds of cities across the U.S. based on perceived risks of mortgage loan defaults.^11^ In addition to the general environmental and economic conditions of neighborhoods, one of the key factors in determining neighborhood risk was the presence of “undesirable” inhabitants, African Americans, or foreign-born individuals. The legacy of redlining lingers, influencing both racial and socioeconomic makeup of communities in present day and shaping structural racism in place.^10–12^

The practice of redlining, however, did not arise from a vacuum nor has its legacy been fixed in time. Ongoing policies and systems have contributed to the evolution of neighborhood socioeconomic and racial composition and characteristics, including the perpetuation of all-white “sundown” towns, the use of restrictive covenants, the development of the interstate highway system, the gentrification of communities, and the selective investment in or displacement of populations ^13–17^. This interplay between historic foundations and the ongoing evolution of policies and practices has created neighborhoods with discreet socioeconomic, housing, and transportation characteristics.

One popular measure of current socioeconomic conditions is the Area Deprivation Index (ADI)^18^. The ADI is a composite measure of 17 different U.S. census variables at the level of census block group. These variables include measures of poverty and wealth, education, employment, housing quality, and housing composition^19^.

The primary objective of this project was to develop a neighborhood classification system (“Neighborhood Trajectories”) that captured both historic redlining as well as current socioeconomic conditions, represented by the ADI. We hypothesized that while many neighborhoods would maintain similar characteristics over time, we would also be able to capture specific locations where socioeconomic conditions may have improved, declined, or remained stable. Furthermore, we hypothesize different Neighborhood Trajectories will have distinct socioeconomic and demographic compositions.

This paper describes the methods and results of the creation of Neighborhood Trajectories using historic HOLC redlining maps and current socioeconomic characteristics available through U.S. census data. This is an adaptable method enabling researchers to choose different socioeconomic endpoints to pursue and develop study-specific Neighborhood Trajectories as a way of describing and capturing neighborhood changes over time. Specific socioeconomic or demographic measures could be used depending on the policy, practice, or evaluation of neighborhood characteristics being evaluated for a given place and time period. We applied this method to describe the Neighborhood Trajectory regional differences and variation of residential redlining to current socioeconomic deprivation between U.S. racial composition of Non-Hispanic/Latino Black residents and Non-Hispanic/Latino White residents.

## Methods

### Block Groups

Our study area was the contiguous United States. We selected the U.S. Census block group as our areal unit of analysis for defining a neighborhood for several reasons. Block groups have relatively small geographic areas with a population range of approximately 600–3000 and it is the unit used by the Area Deprivation Index (ADI). In addition, using the smaller block group polygons rather than a census tract or county, allowed us to capture the areas graded under the HOLC system more precisely.

We obtained block group level population and ethnoracial composition, data from the 2020 decadal U.S. Census and the polygons from IPUMS National Historical Geographic Information System^20^.

### HOLC Grades

**Figure 1:**
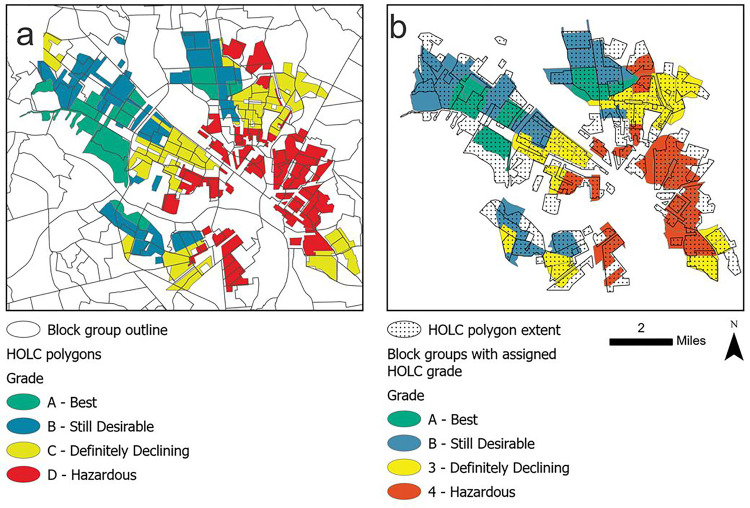
A visual example depicting the assignment of HOLC polygons to block groups Panel a depicts the digitized HOLC polygons in Richmond Virginia overlain with the outlines of block groups. Panel b shows how the HOLC polygons were assigned to block groups. The stippled areas are the extent of the HOLC polygons. Stippled areas with no background color were not assigned to a block group as they covered <50% of a block group. Conversely, solid colors with no stipple are portions of the block groups that were assigned a HOLC grade despite not overlapping a HOLC polygon because >50% of the block group did overlap with the HOLC polygons

The Mapping Inequality: Redlining in New Deal America project digitized HOLC neighborhoods and made the resulting shape files available for download on their website (https://dsl.richmond.edu/panorama/redlining).^21^ We intersected these HOLC neighborhood polygons with the 2020 U.S. Census block group polygons for the entire nation^20^. Most of the block groups were outside of the cities with digitized HOLC polygons and these were removed from our study area. Within the cities in the HOLC program, we wanted to avoid assigning HOLC grades to areas that were developed after the HOLC maps, so we also removed any block group that had less than 50% overlap with a HOLC neighborhood. For the remaining block groups, we assigned a HOLC grade based on the relative proportion of the graded areas in each block group. To do this we assigned a value to each grade: 1 for grade A-Best, 2 for grade B-Still Desirable, 3 for grade C-Definitely Declining and 4 for grade D-Hazardous. We then multiplied the proportion of the graded area in each block group by the assigned value and then summed the products and rounded to the nearest integer which was compared to the allotted values and converted to a HOLC grade for each remaining block group (Fig. 1).

### Area Deprivation Index

The Area Deprivation Index (ADI) is available as national percentiles or state-level deciles.^18, 22^ For our application, we chose the state-level deciles as individual state policies may have influenced how neighborhoods changed over time. The state deciles rank block groups from 1 – Least Deprived to 10-Most Deprived based on a composite of U.S. Census characteristics. Based on the distribution of the counts of block groups in our study area (i.e., block groups that had an assigned HOLC grade), we collapsed the ADI deciles into roughly quartiles of block groups. We named these new ADI categories Least Deprived (deciles 1–2), Less Deprived (deciles 3–5), More Deprived (deciles 6–8), and Most Deprived (deciles 9–10).

### Neighborhood Trajectories

The Neighborhood Trajectories allow us to describe and evaluate changes in neighborhoods from historic HOLC grades to present ADI. To create the Neighborhood Trajectories, the block groups were categorized as “Advantage Stable” for block groups with HOLC grade A-Best and B-Still Desirable and ADI categories of Less or Least Deprived; “Advantage Reduced” for HOLC grade A-Best and B-Still Desirable and ADI categories of More or Most Deprived; “Disadvantage Reduced” for HOLC grade C-Definitely Declining and D-Hazardous and ADI categories of Less or Least Deprived; and “Disadvantage Stable” for HOLC grade C-Definitely Declining and D-Hazardous and ADI categories of More or Most Deprived.

## Results

**Figure 2:**
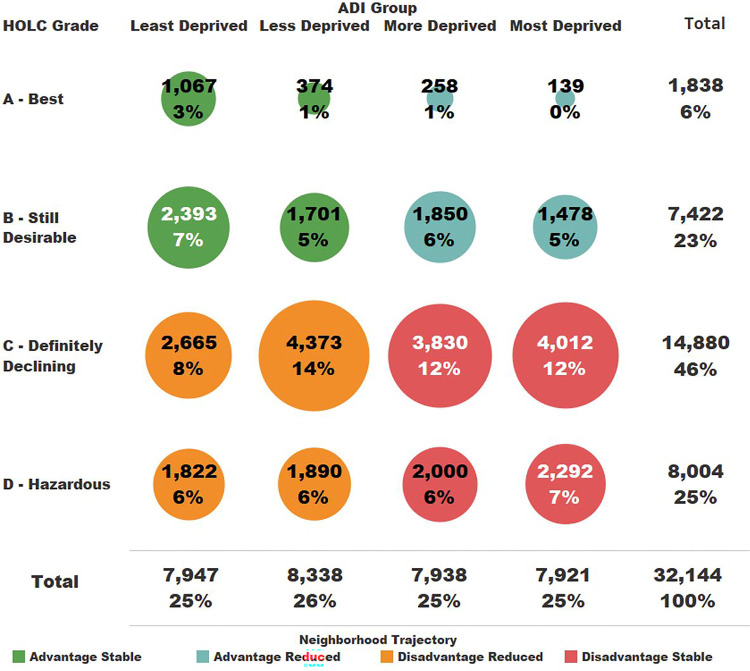
Block group counts by HOLC Grade and ADI group. Dot size is proportional to percentage of block groups and colored based on the Neighborhood Trajectory

There were 241,764 block groups of which 44,330 (18%) overlapped at least partially with a HOLC neighborhood and 32,646 (14%) overlapped at least 50% and therefore met our inclusion criteria. An ADI decile was not assigned to 502 of these block groups due to low population counts and/or high populations residing in group quarters (e.g., dormitories, prisons)^23^, leaving 32,144 (13%) block groups with both an ADI decile and HOLC grade (Fig. 2) in 201 cities across the United States.

Grade C- Definitely Declining had the most block groups (14,880; 46%) while Grade A – Best had the least (1,838; 6%). The ADI Groups ranged between 7,921 (Most Deprived) to 8,338 (Less Deprived) block groups. The largest Neighborhood Trajectories were Disadvantage Stable (12,134; 38%) and Disadvantage Reduced (10,750; 33%) with Advantage Stable (5,535; 17%) and Advantage Reduced (3,725; 12%) representing the Neighborhood Trajectories with the fewest number of block groups (Fig. 2).

**Figure 3:**
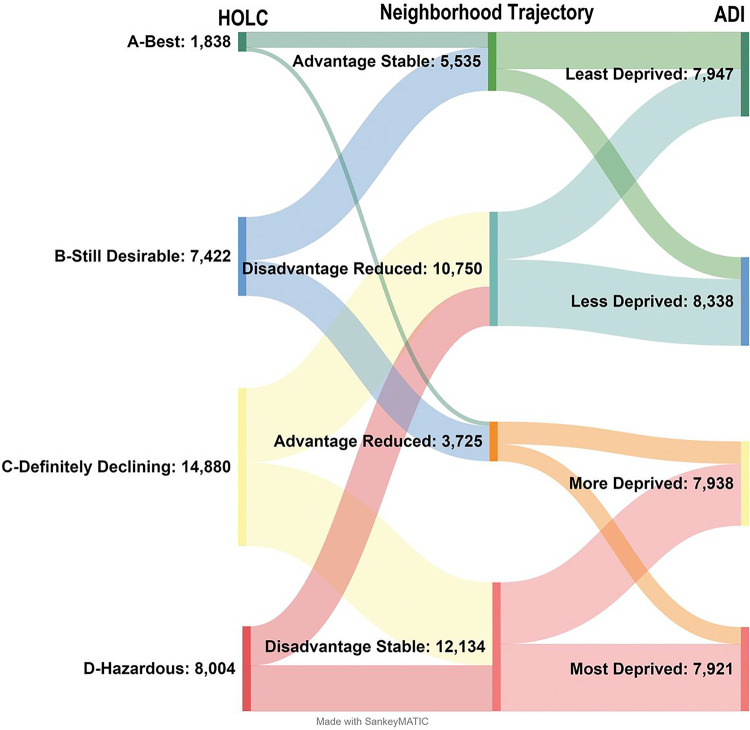
SANKEY chart visualizing the flow of neighborhoods (block group counts) from the historic redlining to today’s ADI

**Figure 4:**
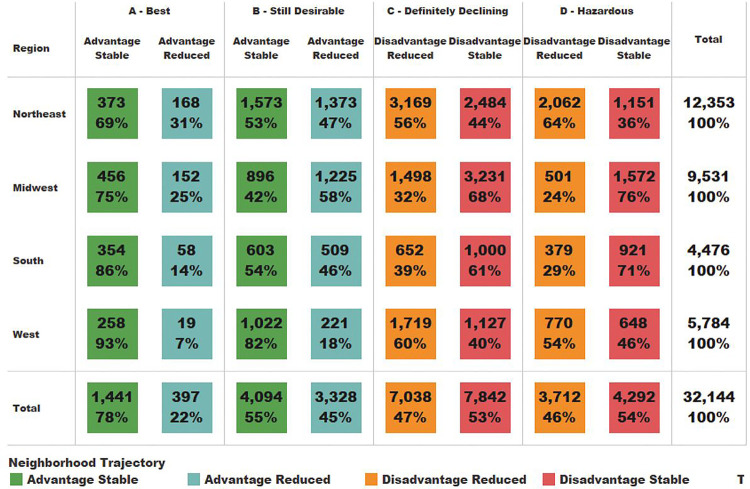
Regional differences in Neighborhood Trajectory block group counts by HOLC grade

The flow of neighborhoods from the 1930’s-40’s HOLC grading system to the contemporary ADI group via the Neighborhood Trajectories is shown in Fig. 3. HOLC grades B-D are roughly equally split between stable and reduced trajectories. However, 78% of Grade A remained Advantage Stable while only 22% are on the Advantage Reduced trajectory. These splits are not consistent across geographic regions (Fig. 4). Overall, HOLC Grade C has 47% of its block groups in the Disadvantage Reduced trajectory and 53% Disadvantage Stable. The proportions are reversed for the Northeast (the region with the most block groups in the study) with 56% Disadvantage Reduced and 44% Disadvantage Stable whereas the neighboring Midwest has only 32% Disadvantage Reduced and 68% remained disadvantaged.

**Figure 5:**
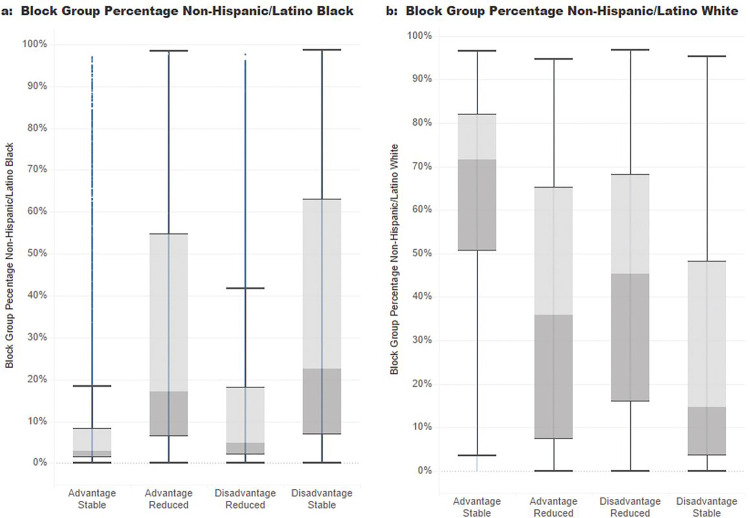
Box plots showing racial composition of block groups by Neighborhood Trajectory. The grey boxes represent the interquartile (25^th^-75^th^ percentile) range with the color changing at the median. The whiskers extend 1.5 times the interquartile range. Any data pointes extending beyond the whiskers are considered outliers

The population living in the study area in 2020 was distributed among the 4 Neighborhood Trajectories similarly to the block group count (Table 1) with Disadvantage Stable containing 38% of the block groups and 36% of the population while the smallest trajectory was Advantage Reduced with 12% of the block groups and 11% of the population. The racial composition of the Neighborhood Trajectories varied from 62% Non-Hispanic/Latino White and 10% Non-Hispanic/Latino Black in Advantage Stable to more similar proportions in Advantage Reduced of 37% Non-Hispanic/Latino White and 31% Non-Hispanic/Latino Black. Variation in block group racial composition differed within Neighborhood Trajectories, notably within the Advantage Stable and Disadvantage Reduced trajectories (Fig. 5).

## Discussion

Structural inequity and racism remain major driving forces behind health inequities yet our ability to capture or measure structural inequity has been challenging. Here we describe one method that captures the dynamic legacy of housing policy. The Neighborhood Trajectories evaluate the influence of the historic policies and practices of residential redlining in the context of ongoing marginalization or development of neighborhoods. Using HOLC maps and current U.S. census bureau data, we established Neighborhood Trajectories for 32,144 block groups across 201 cities in the United States. Of these, most block groups had a trajectory of Disadvantage Stable (38%) or Disadvantage Reduced (33%). However, there was significant geographic variation with the Northeast having a greater proportion of block groups with Disadvantage Reduced compared to the Midwest where the majority of historic disadvantage remained stable. Additionally, we noted distinct patterns of racial/ethnic demographics between each of the four categories, demonstrating how using either historical or current data alone may have failed to capture the unique aspects between block groups. As demonstrated in Fig. 5, for instance, the proportion of White residents in Advantage Stable block groups is much higher than in Disadvantage Reduced, despite each Trajectory having similar current measures of Area Deprivation Index.

Neighborhood Trajectories expand approaches to understanding structural and historic inequalities in the United States. Considering historic features alone as the measure of structural inequity fails to capture the dynamic aspects of ever-evolving policies, practices, and communities. In the context of civil rights in America, historians have described fixed historic factors as having vampiric qualities which “exists outside of time and history, beyond the processes of life and death, [as well as] change and development.”^4^ The Neighborhood Trajectories developed here aim to better classify communities as shaped by both historic factors and the intervening, dynamic changes that happen since that time. As such, our Neighborhood Trajectories used HOLC maps and current census data at the level of the census block group. However, a similar approach could just as easily be used to evaluate policies, practices, or systems, such as evolving environmental regulation or the development of the interstate highway system^24–26^, for example.

Prior work has established a strong association between residential redlining and current outcomes. This includes redlined areas to be associated with increased likelihood of health conditions or access to health care, decreased access to healthy food, and increased exposure to pollution. Additional, extensive work has similarly shown that current neighborhood characteristics are associated with shorter life expectancy, worse outcomes from health care, and worse pedestrian safety.^19, 27–29^ The Neighborhood Trajectory builds on this literature by creating a tool for which dynamic processes and policies that shape current neighborhoods and urban landscapes may be further quantitatively analyzed. Two of the primary challenges of evaluating residential redlining are 1) projecting neighborhood maps that predate present day administrative units (census and municipal) onto current neighborhoods and 2) accounting for or measuring dynamic changes over time. Here we provide one method that bridges HOLC maps with current census boundaries while maintaining fidelity of the original landscape. Prior efforts have tried to translate HOLC grading at the level of census tracts, although this paradigm fails to capture neighborhood heterogeneity at levels smaller than census tract.^30, 31^ Similarly, there are a considerable number of census block groups that overlap with different graded HOLC neighborhoods or with varying degree of areas that are ungraded. We present this method as an approach to use as much information as possible from HOLC maps while avoiding over-attribution of grading to block groups with little area that was graded in HOLC maps. Consequently, we found that 82.2% of HOLC graded areas were captured with Neighborhood Trajectories

This development and use of the Neighborhood Trajectory should be considered in terms of its limitations. First, we only included cities where the Mapping Inequality Redlining in New Deal America^21^ project provided digitized HOLC data. We cannot account for changes that occurred in other cities. Similarly, Neighborhood Trajectories cannot account for socioeconomic and demographic shifts that may have occurred in the unmapped peripheral portions or suburbs of these cities where a considerable degree of additional policies and practices have shaped segregation in the United States, including restrictive covenants.^13–17, 32^

Neighborhood Trajectories describe the area in which people reside, but they do not necessarily describe all residents of an area and they do not track the residents over time who may move into or out of the neighborhood. Likewise, the Neighborhood Trajectories capture the endpoints of historic redlining and current socioeconomic conditions in neighborhoods but do not explain what occurred during the intervening decades. Others have used U.S. Decennial Census data from 1970 to 2010 to categorize the temporal changes in neighborhoods^33, 34^, which allow for a more nuanced analysis, albeit over a shorter time period. Additionally, residential redlining does not capture the full extent of structural racism in the U.S. as there are varying degrees of additional oppressive or segregated pressures including restrictive covenants or sundown towns that shape the present landscape and health.^35^ Similarly, redlining maps did not have uniform impact on communities across the United States. For instance, some residents of redlined areas were prevented from obtaining mortgages at all while other cities had mortgages available for Black residents but restricted the mortgages to properties within redlined areas.^15^

While representing changes from 1930’s to present socioeconomic status, this method does not capture specific or individual policies or practices that could have occurred in neighborhoods over time. Rather, it provides a very high-level perspective of overall trends in cities across the country. Finally, while Neighborhood Trajectories may provide a rough measure of gentrification, with previously disadvantaged communities presently having low deprivation, it does not capture full spectrum of ways in which gentrification could have occurred. Some areas may have experienced equitable investment with uniform improvement of conditions for the community, while other areas may have experienced asymmetric displacement of populations or further segregation within pockets of the community. Here is where evaluation of specific community-specific dynamics will provide important, prescriptive insights to city investment, neighborhood planning, and dismantling of structural racism.

In conclusion, we present one method to capture the dynamic aspect of structural oppression and racism in the United States, from residential redlining to current socioeconomic deprivation. This includes mapping Neighborhood Trajectories for 32,144 block groups in 201 cities in the United States. We believe this method provides a novel approach to evaluating dynamic aspects of structural oppression and racism in the United States. The Neighborhood Trajectories method offers robustness for many research applications that may want to quantify and classify the changes between historic and contemporary socioeconomic to learn more about the temporal trends and impact of historic policies on current neighborhoods.

## Figures and Tables

**Table 1:** Distribution (N; %) of block group population, overall population and racial composition population among Neighborhood Trajectories

	Advantage Stable	AdVantage Reduced	Disadvantage Reduced	Disadvantage Stable	Total
**Block group**	5536 (17%)	3,725 (12%)	10,750 (33%)	12,134 (38%)	32,144 (100%)
**Population**	6,386,644 (17%)	4,023,276 (11%)	13,735,253 (36%)	13,851,600 (36%)	37,996,773 (100%)
Non-Hispanic/Latino Black	620,260 (10%)	1,227,721 (31%)	2,027,080 (15%)	4,279,028 (31%)	8,154,089 (21%)
Non-Hispanic/Latino White	3,961,517 (62%)	1,469,384 (37%)	5,629,159 (41%)	3,533,865 (26%)	1,4593,925 (38%)
